# Meta-analysis of the therapeutic effect of acupuncture and chiropractic on cervical spondylosis radiculopathy: A systematic review and meta-analysis protocol

**DOI:** 10.1097/MD.0000000000018851

**Published:** 2020-01-31

**Authors:** Ping Wang, Guang Zuo, Shuang-qing Du, Tian-ci Gao, Rui-jia Liu, Xiao-zhou Hou, Xu Ji, Jing Yin, Kai-ming Li, Qing Zhang

**Affiliations:** a Department of Education, Wangjing Hospital, China Academy of Chinese Medical Sciences, Beijing; b Hebei Provincial Hospital of Traditional Chinese Medicine; c Department of Bone Injury, Hebei Hospital of Traditional Chinese Medicine, Hebei University of Traditional Chinese Medicine, Hebei; d The First Department of Neurology, Dongzhimen Hospital, Beijing University of Chinese Medicine; e Beijing Tongzhou Integrative Medicine Hospital, Beijing, China.

**Keywords:** acupuncture, chiropractic, cervical spondylotic, systematic review

## Abstract

**Background:**

The pathogenesis of cervical spondylotic is degenerative changes of the cervical intervertebral disc, or bone hyperplasia of the posterior and hook joints, and instability of the joints of the cervical vertebrae. It causes the nerve roots to be stimulated and oppressed. The clinical manifestations are the sensation, movement, and reflex disorder of the cervical spinal nerve roots that are stimulated and oppressed, especially the numbness and pain of the neck, shoulders, upper limbs, and fingers. In this systematic review, we aimed to evaluate the efficacy and safety of acupuncture and chiropractic in the treatment of cervical spondylotic.

**Methods and analysis:**

We will search for PubMed, Cochrane Library, AMED, Embase, WorldSciNet; Nature, Science online and China Journal Full-text Database (CNKI), China Biomedical Literature CD-ROM Database (CBM), and related randomized controlled trials included in the China Resources Database. The time is limited from the construction of the library to September 2019. We will use the criteria provided by Cochrane 5.1.0 for quality assessment and risk assessment of the included studies, and use the RevMan 5.3 and Stata 13.0 software for meta-analysis of the effectiveness, recurrence rate, and symptom scores of cervical spondylotic.

**Ethics and dissemination:**

This systematic review will evaluate the efficacy and safety of acupuncture and chiropractic for cervical spondylotic. Because all of the data used in this systematic review and meta-analysis have been published, this review does not require ethical approval. Furthermore, all data will be analyzed anonymously during the review process trial.

## 1 Introduction

Cervical spondylotic is a clinical syndrome caused by cervical disc degeneration,^[1]^ cervical vertebrae hyperplasia, cervical vertebrae joint and ligament loosening and dislocation stimulation or oppression of cervical nerve roots,^[2–5]^ accounting for 60% of cervical spondylosis 70%, mainly manifested as pain and numbness in the corresponding nerve distribution area. The cause is mostly the protrusion or prolapse of the nucleus pulposus,^[6]^ the bone hyperplasia or traumatic arthritis of the posterior small joint, the formation of the spur of the hook joint,^[7]^ and the loosening and displacement of the adjacent 3 joints can cause the stimulation and oppression of spinal nerve roots.^[8,9]^ The treatment of cervical spondylotic is divided into nonsurgical therapy and surgical therapy.^[10]^ Nonsurgical treatments include continuous (or intermittent) traction of the head and neck, neck circumference braking, and correction of poor posture. Manual massage also has a certain effect,^[11–13]^ but it should be gentle, and should not cause accidents due to rough operation. Surgical treatment is suitable for anterior cervical decompression,^[14,15]^ which is not only effective, but also has little effect on the stability of cervical vertebrae. For patients with vertebral instability or root canal stenosis, intervertebral interfacial internal fixation can also be used to expand and fix the vertebral segments.^[16]^

Acupuncture and chiropractic are important parts of traditional Chinese medicine and have recently been widely used in clinical trials.^[17]^ Recent studies have shown significant effect in reducing chronic pain and tissue fibrosis around the neck area.^[18,19]^ Studies have also shown that acupuncture and chiropractic can accelerate the central nervous system to produce endogenous opioid peptides and activate related receptors by stimulating related acupoints to achieve peripheral analgesia.^[20–22]^ In addition, it can achieve anti-inflammatory effects by increasing the level of β-Ep in inflammatory tissues and serum.^[23]^ Chinese medicine believes that acupuncture and chiropractic can regulate the blood and blood balance of the human body, and the function of the body can also be improved by stimulating acupuncture and chiropractic points. Moreover, it is becoming more and more popular due to its unique advantages of simplicity, convenience, efficacy, and low cost.^[24]^

After preliminary search and database analysis, we found that the frequency of randomized controlled trials (RCTs) of acupuncture and chiropractic treatment in cervical spondylotic is on the rise. Previous clinical trials have shown that acupuncture and chiropractic can reduce pain and improve the quality of life of patients. These effects persist in people with cervical spondylotic. However, due to the limited size and sample size of clinical centers, the current level of evidence-based medical evidence is still insufficient. Therefore, we hope to evaluate the efficacy and safety of acupuncture and chiropractic in the treatment of cervical spondylotic through meta-analysis, to provide sufficient evidence for its clinical application.

## 2 Methods

This systematic review protocol has been registered on PROSPERO CRD42019119941 (https://www.crd.york.ac.uk/prospero/display_record.php?RecordID=119941). The protocol follows the Cochrane Handbook for Systematic Reviews of Interventions and the Preferred Reporting Items for Systematic Reviews and Meta-Analysis Protocol (PRISMA-P) statement guidelines. We will describe the changes in our full review if needed.

### 2.1 Inclusion criteria for study selection

#### 2.1.1 Types of studies

We will gather all studies of acupuncture and chiropractic on treating cervical spondylotic: a systematic review and meta-analysis which, no matter whether they have been published or not, base on the method of RCT. The language is limited to Chinese and English. Non-RCTs, quasi-RCTs, series of case reports, and cross research will be excluded.

#### 2.1.2 Types of participants

1. Compliant diagnostic criteria for cervical spondylotic
2. Imaging examination showed that the cervical disc was prominent, the annulus fibrosus and posterior longitudinal ligament did not rupture, the nucleus pulposus did not escape the annulus fibrosus, and it was consistent with clinical manifestations
3. The height of the intervertebral disc is not <75%

#### 2.1.3 Types of interventions

We will adopt acupuncture and chiropractic treatment of cervical spondylotic as experimental interventions. Considering that the theory of pharmaco-acupuncture, chiropractic, and point injection belongs to another part of TCM, so they will be considered for exclusion.

##### 2.1.3.1 Control interventions

As for control intervention, a person receiving virtual acupuncture and chiropractic treatment can be used as a placebo control, or as a blank control without receiving any treatment. However, once they receive acupuncture and chiropractic combined drugs or other Chinese medicine, the trial will be rejected.

The following treatment comparisons will be studied.

#### 2.1.4 Types of outcome measures

##### 2.1.4.1 Primary outcomes

The main criteria are: visual analog scale for assessing pain levels; computed tomography and nuclear magnetic resonance results; efficacy evaluation method in “Guidelines for Clinical Research of New Drugs in Traditional Chinese Medicine,” score reduction = (pretreatment score − posttreatment score)/pretreatment score × 100%; and intervertebral foramen extrusion test.

##### 2.1.4.2 Secondary outcomes

Secondary assessment criteria include signs and quality of life. At the same time, close attention should be paid to whether adverse reactions or adverse events occur during the experiment to comprehensively evaluate the clinical efficacy and safety of acupuncture and chiropractic in the treatment of cervical spondylotic.

#### 2.1.5 Electronic searches

Database search: Search PubMed, Cochrane, Library, AMED, Embase, WorldSciNet; Nature Science online and China National Knowledge Infrastructure (CNKI), China Biology Medicine disc (CBMdisc). The temporal interval is limited from the time that the databases created to October 2019, and the combination of keyword and free word retrieval is adopted. The search terms include “acupuncture,” “skin acupuncture,” and “cervical spondylotic.” The search term in the Chinese database is the translation of the above word. The complete PubMed search strategy is summarized in Table 1.

**Table 1:**
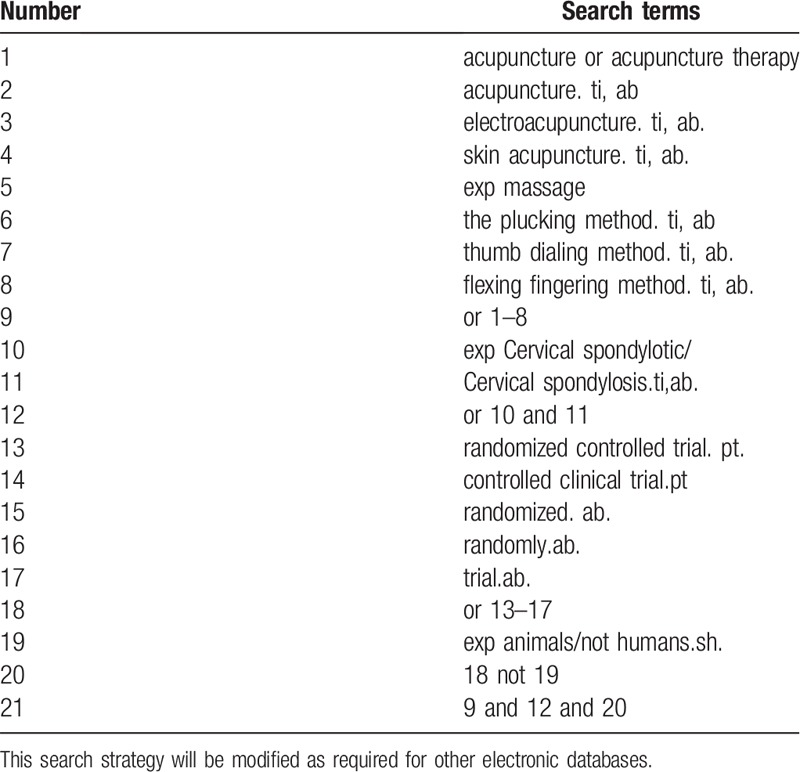
Search strategy used in PubMed database.

#### 2.1.6 Searching other resources

The manual search mainly searched for relevant literatures, earlier than the database above-mentioned, such as “China Rehabilitation Medicine Journal,” “Chinese Acupuncture,” “Chinese Journal of Physical Medicine and Rehabilitation,” “acupuncture and chiropractic Clinical Journal,” and “Chinese Journal of Urology.”

### 2.2 Data collection and analysis

#### 2.2.1 Study identification

**Figure 1:**
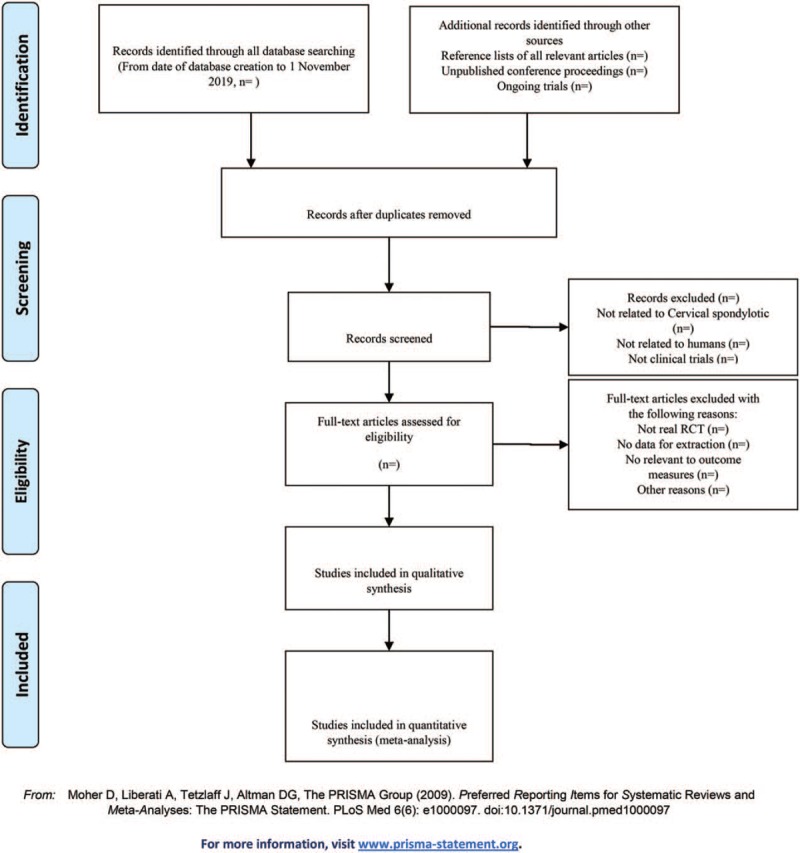
The PRISMA flow chart.

1. There are 2 researchers filtering out the literature that clearly do not conform to the study such as meeting minutes dissertations reviews animal experiments and so on, which, after excluding all the retrieved documents from the duplicated literature, adopt the method of reading the title of the literature, abstracts, etc. The details of selection process will be shown in the PRISMA flow chart (Fig. 1).
2. The 2nd time of screening the literature: Skimming the remaining documents and filtering out unqualified documents such as case reports theoretical discussions and nonconformance of interventions.
3. The 3rd time of screening the literature: Carefully reading the remaining documents and strictly filtering out unqualified documents such as general controlled trials, lacking control group, deficiency of random allocation, incompatible outcome indicator, and the appearance of similar data.
4. As for the literature that cannot be ensured, it would be confirmed by the discussion of the 2 researchers. And if they cannot reach an agreement, the 3rd-party experts would get involved, which aims at absorbing the appropriate RCTs into the study.

#### 2.2.2 Data extraction and management

The literature data extraction will be completed independently by 2 researchers and the data form uniformly developed by the researcher was filled out. The data extraction content includes the following:

1. General information: article title, first author, corresponding author, time of publication research, evaluation correspondence, contact information.
2. Research method: design pattern, ample size, random allocation, random hiding, blind method, baseline level.
3. Participants: Patients age, gender, cervical spondylotic diagnostic criteria, severity, ethnicity study, location.
4. Intervention: acupuncture, acupuncture and chiropractic point, period of treatment, treatment frequency.
5. Efficacy evaluation: main observation indicators secondary observation indicators safety indicators and number of adverse reactions.
6. Note: sources of funds, medical ethics audit, important references.

#### 2.2.3 Assessment of risk of bias in included studies

As for the literature quality evaluation, we will use the bias risk assessment tool recommended by Cochrane to assess the quality of all included literature and risk of bias. The assessment include: sequence generation; allocation concealment; blinding of participants, personnel, and outcome assessors; incomplete outcome data; selective outcome reporting; other sources of bias. The evaluation above would be independently evaluated by 2 researchers. If there are different opinions, we discuss them. If there are still differences exist, we would consult the 3rd appraiser. Otherwise, we need to consult with the Cochrane Professional Group for solution.

#### 2.2.4 Statistical analysis

The meta-analysis studied in this review will adopt RevMan 5.3 and Stata 13.0 statistical software. Heterogeneity test will be used for the inclusion of the study, and random or fixed effect models will be adopted, with *P* < .05 as the test standard. If the heterogeneity between the results is too large, the random-effects model (REM), which deduce the source of heterogeneity by sensitivity analysis, will be used for the rest analysis. Secondly, according to the different type of statistical data, the binary categorical variable will use the odds ratio and its 95% confidence interval (CI) as the effect analysis index. As for the continuous variable, the standardized mean difference and its 95% CI will be used as the effect analysis index. If the outcome measures only provide the means and standards deviation before or after treatment, the Mean_change_ and the SD_change_ are obtained according to the method provided in Cochrane Handbook 5.1.0.

The forest map and funnel plot were drawn and analyzed using RevMan 5.3 software, and the funnel plot was used to analyze potential publication bias. As for the literature quality evaluation, we will use the bias risk assessment tool recommended by Cochrane to assess the quality of all included literature and risk of bias. The assessment include: sequence generation; allocation concealment; blinding of participants, personnel, and outcome assessors; incomplete outcome data; selective outcome reporting; other sources of bias. The evaluation above would be independently evaluated by 2 researchers. If there are different opinions, we discuss them. If there are still differences exist, we would consult the 3rd appraiser. Otherwise, we need to consult with the Cochrane Professional Group for solution.

#### 2.2.5 Assessment of heterogeneity

If there is significant heterogeneity between group of studies, we will explore the reasons for the existence of heterogeneity from various aspects, such as the characteristics of the subjects and the degree of variation of the interventions. Necessarily, sensitivity analysis or subgroup analysis would be adopted to explain the heterogeneity.

#### 2.2.6 Sensitivity analysis

We will conduct a sensitivity analysis to identify whether the conclusions are robust in the review according to the following criteria: sample size, heterogeneity qualities, and statistical model (REM or fixed-effects model).

#### 2.2.7 Publication bias

If a result of a meta-analysis contains more than 10 articles and above, we will use a funnel plot to test the risk of publication bias.

#### 2.2.8 Quality of evidence

The quality of evidence for the main outcomes will also be assessed with the GRADE approach. The evaluation included bias risk; heterogeneity; indirectness; imprecision; publication bias. And each level of evidence will be made “very low,” “low,” erate,” or “high” judgment.

## 3 Discussion

In recent years, the clinical RCT of cervical spondylotic has been increasing, but it is still unsatisfactory in the diagnosis and treatment of diseases.^[25]^ Clinicians have not yet reached a consensus on the treatment principles and assessment of the disease, and lack uniform standardization standards.^[26]^ At present, there has not been a large-scale epidemiologic investigation of the disease, and there are few reports in the literature. Traditional Chinese medicine has a profound theoretical foundation and rich clinical experience in the treatment of cervical spondylotic.^[27]^ Acupuncture and chiropractic are the indispensable part of Chinese medicine. It has the characteristics of small side effects and easy operation. It has been used to treat various systemic diseases such as diarrhea and cervical spondylosis. The therapy mainly regulates the balance of blood and blood by stimulating the acupuncture and chiropractic points of the human body, and achieves the effect of balancing yin and yang. Although the specific mechanism of acupuncture and chiropractic treatment of cervical spondylotic remains unclear, clinical studies have shown that acupuncture and chiropractic treatment of cervical spondylotic can relieve pain and alleviate symptoms. To the best of our knowledge, there is no comparability between the efficacy of acupuncture and chiropractic in the treatment of cervical spondylotic.

Therefore, we will use a systematic review and meta-analysis to evaluate the efficacy and safety of acupuncture and chiropractic in the treatment of cervical spondylotic.^[28]^ The results of this study may provide a possible ranking for acupuncture and chiropractic treatment of cervical spondylotic. In addition, the scoring method will be used to assess the quality of the evidence for the primary outcome. We hope that these results will provide clinicians with the basis for acupuncture and chiropractic treatment of cervical spondylotic and provide the best choice for patient care. In addition, although this study will conduct a comprehensive search, it will not search for languages other than Chinese and English, which will lead to some bias.

## Author contributions

**Data curation:** Ping Wang, Xiao-zhou Hou.

**Formal analysis:** Kai-ming Li, Guang Zuo, Tian-ci Gao.

**Funding acquisition:** Qing Zhang, Rui-jia Liu.

**Project administration:** Xu Ji, Kai-ming Li.

**Supervision:** Xiao-zhou Hou, Guang Zuo.

**Validation:** Ping Wang.

**Writing – original draft:** Qing Zhang, Ping Wang.

**Writing – review & editing:** Rui-jia Liu, Ping Wang.
